# A Differential Innate Immune Response in Active and Chronic Stages of Bovine Infectious Digital Dermatitis

**DOI:** 10.3389/fmicb.2018.01586

**Published:** 2018-07-19

**Authors:** Kaitlyn M. Watts, Cristina Fodor, Caroline Beninger, Priyoshi Lahiri, Rakel Arrazuria, Jeroen De Buck, Cameron G. Knight, Karin Orsel, Herman W. Barkema, Eduardo R. Cobo

**Affiliations:** ^1^Department of Production Animal Health, Faculty of Veterinary Medicine, University of Calgary, Calgary, AB, Canada; ^2^Bachelor of Health Sciences, Cumming School of Medicine, University of Calgary, Calgary, AB, Canada; ^3^Department of Veterinary Clinical and Diagnostic Sciences, Faculty of Veterinary Medicine, University of Calgary, Calgary, AB, Canada

**Keywords:** *Treponema* spp., digital dermatitis, cattle, keratinocytes, cathelicidins, β-defensins, cytokines

## Abstract

Digital dermatitis (DD) commonly associated with *Treponema* spp. infection is a prevalent infectious bovine foot disease characterized by ulcerative and necrotic lesions. Lesions associated with DD are often classified using the M-stage scoring system, with M0 indicating healthy heel skin and M4 indicating chronic lesions. Current treatments utilizing antimicrobials or chemical footbaths are often ineffective and rarely cure DD lesions. Understanding the function of the innate immune response in the pathogenesis of DD will help to identify novel therapeutic approaches. In this study, the expression of the local innate host defense peptides cathelicidins and β-defensins was investigated in cows with DD and associated with the presence of treponemes and inflammatory reactions. Samples from active ulcerative DD lesions (M2) had considerable epidermal neutrophilic infiltration and increased gene expression of β-defensin tracheal antimicrobial peptides compared to control skin. Samples from acute lesions also had elevated local Cxcl-8 and TLR4 gene expression and abundant treponemes as identified by direct visualization, immunohistochemistry, and culture. Conversely, the anti-inflammatory peptide IL-10 was elevated in skin from chronic (M4) lesions, whereas bovine cathelicidin myeloid antimicrobial peptide 28 (Bmap-28) was increased in skin from oxytetracycline-treated M2 lesions. Experiments using cultured human keratinocytes challenged with *Treponema* spp. isolated from clinical cases of bovine DD showed that structural products from treponemes are able to initiate the innate immune response, in part through TLR2 signaling. These findings indicate that neutrophil influx, Cxcl-8, and β-defensin are key markers of active DD. Cathelicidins and IL-10 seem important in response to treatment or during the chronic proliferative stages of the disease.

## Introduction

Bovine digital dermatitis (DD) is an infectious disease of cattle characterized by ulcerative and necrotizing foot lesions. The pain and lameness caused by DD are among the cattle industry’s most prominent animal welfare concerns. The negative effects of DD include reduced milk production, lower reproductive rates, premature culling, and weight loss (Cha et al., 2010; Evans et al., 2016). Thus, since its first identification in Italy in 1974, DD has emerged as the leading cause of lameness in the cattle industry worldwide with considerable costs associated with its treatment and management (Solano et al., 2016; Jacobs et al., 2017; Orsel et al., 2017).

Digital dermatitis is a polymicrobial disease (Zinicola et al., 2015); however, the fastidious, anaerobic, and highly motile spirochetes of the genus *Treponema* (collectively referred to as treponemes) are suspected to be the primary causative agents (Dopfer et al., 1997). Various *Treponema* spp. are consistently detected in active bovine DD lesions and are often absent from healthy feet (Walker et al., 1995; Dopfer et al., 2012; Krull et al., 2014). However, the cause of DD has not yet been definitively identified (Zinicola et al., 2015; Krull et al., 2016).

Treatment of DD typically involves the use of topical antibiotics and chemical footbaths (Solano et al., 2015). These approaches are often ineffective, usually require repeated applications, and are potentially hazardous for humans, animals, and the environment (carcinogenic chemicals, antimicrobial resistance, food safety) (Orsel et al., 2017).

Lesions typically occur in the skin of the palmar/plantar interdigital bulb (and sometimes around the coronary band), usually on the hind feet (Dopfer et al., 1997). Lesions are clinically classified into 6 M stages based on their macroscopic appearance: M0, normal skin; M1, formation of a small acute ulcerative area <2 cm in diameter; M2, a larger and acute ulcerative lesion >2 cm in diameter; M3, a healing lesion; M4, a chronic or recurrent lesion (Dopfer et al., 1997); and M4.1, an added sixth stage characterized by an M4 lesion combined with a painful and active M1 focus (Berry et al., 2012). Stages M1, M2, and M4.1 are commonly associated with acute inflammatory processes, whereas M4 is associated with chronic disease. When DD M2 lesions are treated (for instance, topically with antibiotics), they may remain as active M2 lesions or may heal and progress to the M3 stage. Although DD M stages are numerically ordered, they do not necessarily progress chronologically and any lesion may take on any M stage. Histologic characterization of the different M stages has been somewhat limited and contentious, but histologic lesions such as hyperkeratosis, necrosuppurative epidermitis, and necrotizing vasculitis have been described (Dopfer et al., 1997; Krull et al., 2016).

While the causes of, and inflammatory responses associated with, DD have been studied, the role of innate immunity in the bovine skin as it pertains to the establishment of DD is largely unknown. In human studies, *T. denticola* has been shown to stimulate gingival epithelial cells via toll-like receptors (TLRs) 2 (Ruby et al., 2007). Likewise, in mice, major outer sheath proteins and lipopolysaccharides of *T. denticola* have induced expression of inflammatory mediators via TLR2 and TLR4 in macrophages (Nussbaum et al., 2009). However, little is known about the involvement of Tlrs in the bovine skin. Of particular interest in the innate immune response are the host defense peptides. Cathelicidins and β-defensins are evolutionarily conserved amphipathic cationic host defense peptides with innate antimicrobial and immunomodulatory functions (Zasloff, 2002). Cathelicidins and β-defensins are secreted by skin epithelial cells and neutrophils and, when induced by invading pathogens, are able to promote bacterial killing and neutrophil recruitment (Zasloff, 2002). While only a single cathelicidin is expressed in humans (cathelicidin antimicrobial peptide, CAMP) and in mice (cathelicidin-related-antimicrobial-peptide; Cramp), cattle uniquely express at least 8 cathelicidin genes (Scocchi et al., 1997; Tomasinsig and Zanetti, 2005; Young-Speirs et al., 2018). The cathelicidin bovine myeloid antimicrobial peptide (Bmap) 28 has been shown to have antimicrobial activity in mammary epithelial cells (Tomasinsig et al., 2010). Humans and cattle produce several types of β-defensins. β-defensin 2 is the most inducible in humans (Schneider et al., 2005) whereas tracheal antimicrobial peptide (Tap) (Diamond et al., 1991) and lingual antimicrobial peptide (Lap) are the key β-defensins in the gut and udder epithelium of cattle (Schonwetter et al., 1995). To gain insights into the skin innate immune response during the various stages of DD infection, we investigated the expression of cytokines, host defense peptides, and Tlrs in cattle with active and chronic DD lesions. We also further explored the innate immune response to DD infection using cultured keratinocytes exposed to treponemes isolated from cows with DD.

## Materials and Methods

### Collection of Biopsies From Foot Lesions in Cattle

All studies were carried out in accordance with the recommendations of the University of Calgary Animal Care Committee. The protocol (AC16-0070) was approved by the University of Calgary Animal Care Committee. All the animal owners gave written informed consent for the use of animals.

With the assistance of certified hoof trimmers, adult cows from dairy farms in Alberta, Canada were screened for DD, classified using the 6 M-stage classification system (Dopfer et al., 1997), and utilized for skin biopsies (M1, *n* = 22; M2, *n* = 54; M4, *n* = 8; M4.1, *n* = 14). Of the 54 cows with M2 foot lesions, 24 had been treated topically (Tx-M2) 7 days before sampling with oxytetracycline (DIN 00719315; Bio Agri Mix, Mitchell, ON, Canada) and bandaged (Berry et al., 2010). Lesions were sampled using a 4-mm biopsy punch (Miltex, Integra Life Sciences Corporation, York, PA, United States) after intradermal injection of 2 mL of lidocaine for local anesthesia. Two biopsy samples were taken. One sample was divided into two for (1) inoculation in RNA*later* stabilization solution (AM7021; Thermo Fisher Scientific, Waltham, MA, United States) for gene expression analysis and (2) fixation in 10% formalin for histologic evaluation. The other whole biopsy sample was placed immediately into anaerobic transport media (AS-911; Anaerobe Systems, Morgan Hill, CA, United States) and transported within 8 h at room temperature to the laboratory at the University of Calgary for direct visualization of treponemes and culturing. Biopsy samples from the bulb of the heel were obtained from cattle with no DD lesions (M0, *n* = 24) within 2 h of death at an Alberta slaughterhouse.

### Histopathologic Scoring

Tissue sections were fixed in 10% neutral buffered formalin and processed routinely for histologic examination. Formalin-fixed, paraffin-embedded sections were cut at a thickness of 5 μm and stained using haematoxylin and eosin (H&E). Slides were examined by a veterinary pathologist and scored according to the three-tiered Iowa Digital Dermatitis Staging System (IDDSS) (Krull et al., 2016). Briefly, Grade 1 represents normal bovine skin; Grade 2 is hyperkeratotic and acanthotic with surface hemorrhage and erythrocytic crusts; and Grade 3 has segmental, localized, necrotizing to necrosuppurative epidermitis. Grade 3 lesions may also include individual epidermal cell necrosis, ballooning degeneration of epidermal cells, necrotizing vasculitis, and intralesional bacteria consisting of spirochetes, bacilli, and coccobacilli.

### *Treponema* Culture and Identification in Bovine Skin Lesions

A subset of 45 biopsy samples inoculated into anaerobic transport media was aseptically cut into longitudinal sections using a sterile No. 10 scalpel within an anaerobic cabinet (5% CO_2_, 5% H_2_, balanced nitrogen). Biopsy fragments were directly visualized for the presence of treponemes using darkfield microscopy based on morphological characteristics (thin corkscrew-shaped bacteria between 0.1 and 0.4 μm in width and 4–15 μm in length) and motility pattern (rotation about their longitudinal axis) (Radolf, 1996). For treponeme culturing, a tissue section from each biopsy sample was macerated in a sterile Petri dish and inoculated into oral treponeme enrichment broth (OTEB) (AS-603; Anaerobe Systems) supplemented with 5 μg/mL of enrofloxacin, 5 μg/mL of rifampicin, and 10% bovine serum (Gomez et al., 2012). Samples were incubated for up to 10 days at 37°C in the anaerobic cabinet and the presence of treponemes was confirmed by direct visualization as above.

Immunohistochemistry and silver-staining (Dopfer et al., 1997) techniques were used to identify treponemes within paraffin-embedded skin samples to histologically confirm the presence of treponemes within the DD lesions. Silver-staining was conducted as previously described (Dopfer et al., 1997). For immunohistochemistry assay, paraffin-embedded tissues were sectioned, deparaffinized with xylene, and blocked with 3% hydrogen peroxide, and 2% bovine serum albumin. Slides were incubated with a rabbit polyclonal anti-treponeme antibody (1:100, overnight), washed with phosphate-buffered saline (3×) and probed with a goat anti-rabbit antibody conjugated to peroxidase (1:1,000, 1 h). After a second washing step, antibody localization was determined using 3,3-diaminobenzidine (Sigma-Aldrich, Oakville, ON, Canada) and observed under a light microscope. The rabbit polyclonal anti-treponeme antibody was obtained by inoculating into rabbits a mixture of *Treponema* spp. isolated from DD inactivated by sonication and 10% neutral buffered formalin, and emulsified in Freud’s complete adjuvant. The antisera were tested for reactivity by Western blotting against the *Treponema* spp. outer membrane proteins and by *Treponema* spp. immunofluorescence (data not shown).

### Keratinocyte Culture

To further explore the pathogenesis of DD and characterize the direct effects of treponemes on the epidermis, a spontaneously transformed aneuploid immortal keratinocyte cell line from adult human skin (HaCaT; kindly provided by Dr. R. Gallo, University of California, San Diego, CA, United States) was used as an *in vitro* skin model. Whereas recently developed primary bovine skin keratinocytes isolated from biopsy samples seem promising for short-term studies (Evans et al., 2014), HaCaT cells have widely used with a more consistent gene transcription of host defense peptides in response to cytokines than primary human keratinocytes (Seo et al., 2012). HaCaT cultures were maintained in DMEM/F12 media (LS12500062; Gibco, Thermo Fisher Scientific) with 10% FBS (A3160702; Gibco, Thermo Fisher Scientific), 1% sodium pyruvate (1 mM; 11360070; Thermo Fisher Scientific), 7.5% sodium bicarbonate (1 mM; 25080094; Thermo Fisher Scientific), L-glutamine, and penicillin (100 U mL^-1^), and streptomycin (100 μg mL^-1^; 15140122, Thermo Fisher Scientific). Cells were grown in a humidified environment of 95% air and 5% CO_2_ at 37°C.

### *Treponema* Challenge to Cultured Keratinocytes

Cultured HaCaT cells were exposed to treponemes isolated from the skin lesions of cows diagnosed with DD and cultured as indicated above. Cells were incubated for 2 and 24 h under aerobic conditions with each of the following: (1) live treponemes concentrations [up to 1 × 10^8^ colony forming units (CFU)/mL]; (2) lysed (sonicated) structural *Treponema* proteins; and (3) soluble factors secreted by treponemes.

For production of treponeme structural proteins and soluble factors, bacterial *Treponema* spp. cultures containing approximately 3.4 × 10^7^ CFU/mL in 6 mL were harvested with an OD_550 nm_ > 0.05 (where OD_550 nm_ 0.1 = 1 × 10^7^ CFU/mL), pelleted by centrifugation (7,500 rpm; 10 min) and re-suspended in Dulbecco’s modified Eagle’s medium F12 1:1 (DMEM/F12; Gibco, Thermo Fisher Scientific). Bacteria were pelleted (7,500 rpm; 10 min), washed twice in sterile phosphate-buffered saline (LS10010031; Gibco, Thermo Fisher Scientific), and re-suspended in DMEM/F12 media prior to sonication (30% amplitude; 30-s intervals; 15 s off for a total of 7 min). Disruption of bacterial integrity was confirmed by dark-field microscopy and subsequent lack of growth when cultured. Bacterial proteins were quantified using a BCA assay (Pierce BCA protein assay kit; 5000201; Thermo Fisher Scientific).

For the production of soluble factors secreted by treponemes, supernatants from cultured treponemes incubated in DMEM/F12 media were collected after 2 h in anaerobic conditions. For this, bacteria were pelleted and the supernatant was confirmed free of bacteria by dark-field microscopy and subsequent lack of growth when cultured. HaCaT cell viability was assessed after the treponeme challenges via trypan blue staining and enumeration under a light microscope to evaluate the effect of bacterial exposure on keratinocyte survival.

### Evaluation of Gene Expression

Transcriptional relative messenger gene (mRNA) expression of Cxcl-8, IL-10, the β-defensins, Tap and Lap, and cathelicidin Bmap-28 in bovine foot skin, and of IFN-γ, Cxcl-8, IL-10, TLR2, and TLR4 in human keratinocytes, was quantified by real-time reverse transcription quantitative polymerase chain reaction (RT-qPCR). Note that the HUGO Gene Nomenclature Committee guidelines were used to name protein coding bovine and human genes^1^. Samples of total RNA from the bovine skin and human keratinocytes were isolated using an RNA tissue kit (E.Z.N.A Tissue RNA Kit; R6688-01; Omega Bio-Tek, Norcross, GA, United States) and TRIzol reagent (15596018; Invitrogen, Thermo Fisher Scientific), respectively, according to the manufacturers’ instructions. Complementary DNA (cDNA) was prepared from 1 μg of total RNA using Moloney murine leukemia virus reverse transcriptase (qScript cDNA synthesis kit; 101414-098 Quantabio, Beverly, MA, United States). The quality and quantity of the resulting RNA and cDNA were determined using a NanoVue Spectrophotometer (GE Healthcare). The absence of contaminating genomic DNA from RNA preparations was verified using a minus-reverse transcriptase control (i.e., a sample with all RT-qPCR reagents except reverse transcriptase). RT-qPCR was performed using a CFX-96 real-time PCR system (Bio-Rad, Mississauga, ON, Canada). Each reaction mixture contained 100 ng of cDNA, 1× SsoAdvanced Universal SYBR Green Supermix (1725270; Bio-Rad) and 0.5 μM of each specific primer, in a final volume of 10 μL. Bovine primers for Tap, Lap, CxcL-8, IL-10, and β-actin were obtained from previously reported sequences (**Table 1**). Human primers for CAMP (PPH09430A), Cxcl-8 (PPH00568A), IL-1β (PPH00171C), TNFα (PPH00341F), IFN-γ (PPH00380C), IL-10 (PPH00572C), TLR2 (PPH01808A), TLR4 (PPH01795F), DEFB4A (PPH11010A), and glyceraldehyde-3-phosphate dehydrogenase (GAPDH) (PPH00150F) were obtained from a commercial source (RT^2^ qPCR Primer Assay; Qiagen, Toronto, ON, Canada). The primers used in this study were experimentally verified for specificity and efficiency. The RT-qPCR efficiency for each gene was calculated from the slope and determined by a linear regression model according to the equation: Efficiency = 10 × (-1/slope)-1, as indicated in the MIQE Guidelines (Bustin et al., 2009). *R*^2^ values were also calculated and were >95%. Reaction mixtures were incubated at 95°C for 5 min, followed by denaturation for 5 s at 95°C and combined annealing/extension for 10 s at 60°C for a total of 40 cycles. Two housekeeping genes, β-actin and GAPDH, were used to standardize bovine and human samples, respectively. Negative controls for cDNA synthesis and PCR procedures were included in all cases. Target mRNA values were corrected relative to the housekeeping genes. Data were analyzed using the 2^-ΔΔ*CT*^ method. Results are reported as mean fold changes of target gene transcription levels in infected groups versus the uninfected control group.

**Table 1:** Details of primers used for mRNA relative quantification by qRT-PCR in bovine foot skin samples.

Gene	Primer sequence	Accession #	Annealing temp (°C)	Reference
IL-8	F: GTTGCTCTCTTGGCAGCTTT	NM_173925.2	60	Refaai et al., 2013
	R: GGTGGAAAGGTGTGGAATGT			
IL-10	F: TGTATCCACTTGCCAACCAG	NM_174088.1	60	Refaai et al., 2013
	R: CAGCAGAGACTGGGTCAACA			
IFN-γ	F: TTCTTGAATGGCAGCTCTGA	NM_174086.1	60	Refaai et al., 2013
	R: TTCTCTTCCGGCTTTCTGAGG			
TLR2	F: TGGAATTAAGCCATGATGTCAA	XM_019978020.1	60	Jann et al., 2008
	R: GACCACCACCAGACCAAGAC			
TLR4	F: ACCCACCTCTCCACCTTGTACTG	XM_019966825.1	60	Herath et al., 2006
	R: CCAGCCAGACCTTGAATACAGG			
GAPDH	F: GGGTCATCATCTCTGACCT	NM_001034034.1	60	Refaai et al., 2013
	R: GGTCATAAGTCCCTCCACGA			
β-actin	F: CAAGGACCTCTACGCCAAC	NM 173979.3	60	Herath et al., 2006
	R: AGAAGCATTTGCGGTGGAC			


### Statistical Analyses

Normality was checked using the Shapiro–Wilk test (Royston, 1982). Normally distributed (parametric) results are graphed as means and bars represent SEM from a minimum of two independent experiments, each one performed in duplicate or triplicate. All comparisons were performed by one-way ANOVA using Tukey’s *post hoc* test. The relationship between M-stages and the presence of treponemes was assessed by the non-parametric Fisher’s exact test. *P*-values of <0.05 were considered statistically significant. All statistical analyses were performed with Graph Pad Prism software (Graph Pad 5.0; La Jolla, CA, United States).

## Results

### Active Bovine DD Lesions Were Ulcerated and Had Severe Neutrophilic Inflammation

**FIGURE 1:**
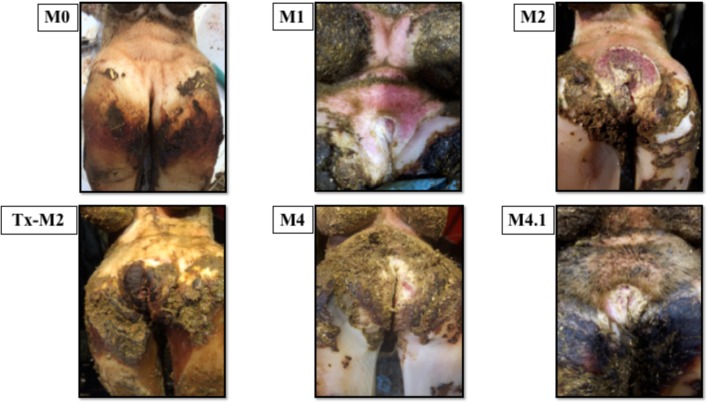
Macroscopic skin lesions in the feet of cattle with different M grades of clinical digital dermatitis. M0: normal healthy skin; M1: small ulcerative lesion <2 cm in diameter; M2: ulcerative lesion >2 cm in diameter with a “strawberry” like appearance; Tx-M2: lesions treated with topical oxytetracycline antibiotics for 7 days prior to collection; M4: chronic lesions with occasional epidermal proliferation; M4.1: M4 lesion with small, central active lesion.

**FIGURE 2:**
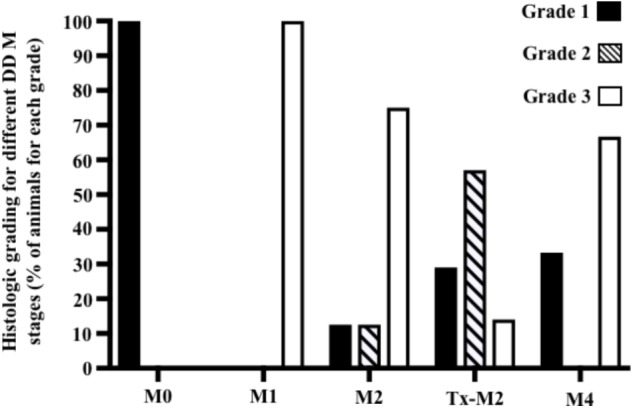
Graphical representation of histological grading scores observed for foot lesions of cows at different M stages of digital dermatitis. Lesions were graded histologically according to the Iowa Digital Dermatitis Staging System (Krull et al., 2016). Grade 1 = normal skin; grade 2 = intermediate lesions; grade 3 = most severe lesions. The M scoring system is described in **Figure 1**.

Cows without clinical DD foot lesions (M0) were all identified as having histopathologically normal heel skin (IDDSS Grade 1) and no treponemes were detected by immunohistochemistry. All cows with M1 lesions, characterized by skin ulceration (<2 cm in diameter) in the palmar or plantar interdigital space (**Figure 1**), had severe inflammation (IDDSS Grade 3), although with less hyperkeratosis and infiltration of leukocytes than M2 lesions (**Figure 2**). Most of the cows (80%) with M2 lesions, characterized by ulcerated (“strawberry-like”) interdigital lesions (>2 cm in diameter; **Figure 1**), also had the most severe histologic lesions (IDDSS Grade 3; **Figure 2**). The Tx-M2 cows displayed the greatest diversity in gross lesion appearance, with lesions ranging from ulcerated/acute to resolved/healing (**Figure 1**). Histologically, Tx-M2 lesions were categorized into all three IDDSS grades, with no clear pattern observed (**Figure 2**). Likewise, the histologic grades of M4 lesions were also variable, with some classified as histologically normal skin (IDDSS Grade 1; 30% of the cows) and others as severe lesions (IDDSS Grade 3; 70% of the cows; **Figure 2**) with macroscopically thickened solid white papillomatous proliferations (**Figure 1**). M4.1 lesions showed reactivation of resolving lesions with small (<2 cm diameter) acutely ulcerated centers (**Figure 1**).

**FIGURE 3:**
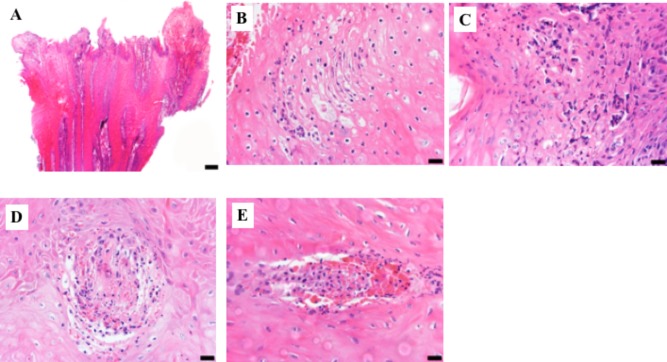
Representative histologic lesions from biopsy samples graded as 3 using the Iowa Digital Dermatitis Staging System (Krull et al., 2016). Hematoxylin and eosin stain. **(A)** Low magnification photograph of epidermal surface showing acanthosis with superficial epidermal necrosis and hemorrhage. Bar = 200 μm. **(B)** Superficial epidermis showing ballooning degeneration, individual cell necrosis, and a predominantly neutrophilic infiltrate. Bar = 20 μm. **(C)** Superficial necrotizing epidermitis with a severe neutrophilic infiltrate. Bar = 20 μm. **(D,E)**. Two foci of superficial epidermal vasculitis with associated hemorrhage and neutrophilic inflammation. Bar = 20 μm.

The severe inflammation observed in M2 and M1 lesions, classified as IDDSS Grade 3, was associated with local dermatitis and the presence of neutrophils, necrosis, “ballooning” epithelial degeneration (**Figures 3A–C**) and spirochete bacteria, as detected by both, silver staining and immunohistochemistry (**Supplementary Figures S1A–C**). Additionally, IDSSS Grade 3 lesions had superficial dermal vasculitis accompanied by hemorrhage and neutrophilic infiltration (**Figures 3D,E**). Taken together, DD M1 and M2 lesions displayed severe acute ulcerative dermatitis with an accumulation of inflammatory cells, particularly neutrophils. Lesions in the Tx-M2 and M4 DD stages showed less inflammation and some healing characteristics. Overall, not all macroscopical M scores were correlated with a single skin histopathological response (IDDSS grade).

### Treponemes Were Mostly Isolated From Active Bovine DD Lesions

**FIGURE 4:**
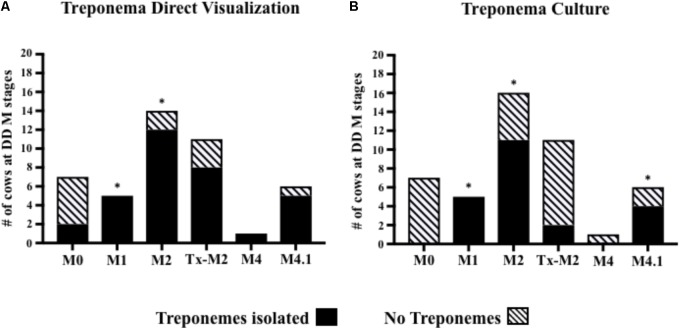
Number of cows at different M stages of digital dermatitis from which treponemes were isolated from foot lesions by direct visualization **(A)** or culture **(B)**. ^∗^*P* < 0.05 was considered significant for the relationship between M-stages and treponemes identification (Fisher’s exact test). The M scoring system is described in **Figure 1**.

Treponemes were detected by direct visualization more frequently in cows with M1 (100%) and M2 (85%) lesions (*P* < 0.05) than in cows with normal skin (**Figure 4A**). Likewise, treponemes were often cultured from cows with active lesions [M1 (100%), M2 (68%), and M4.1 (66%)] compared with cows with normal skin (*P* < 0.05; **Figure 4B**). The presence of treponemes in M2 lesions was confirmed by immunohistochemistry and silver-staining, which revealed aggregates of spirochete bacteria in the ulcerated epidermal surfaces (**Supplementary Figures S1A–C**; arrows). Most of the Tx-M2 cows (85%) had treponemes in their lesions after treatment as detected by direct visualization (**Figure 4A**); however, treponemes were successfully cultured from only 18% of the samples from these animals (**Figure 4B**).

### In Active Ulcerative Bovine DD Lesions Host β-Defensins Were Transcriptionally Upregulated, Whereas the Cathelicidin Bmap 28 Was Downregulated

**FIGURE 5:**
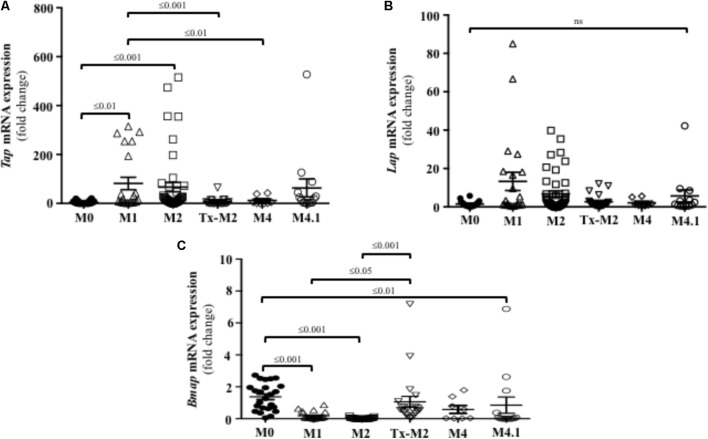
Transcriptional gene expression of the β-defensins Tap **(A)** and Lap **(B)** and the cathelicidin Bmap-28 **(C)** in foot lesions of cows at different M stages of digital dermatitis. Relative mRNA gene expression was determined by RT-qPCR. Means + SEM are shown. *P* < 0.05 was considered significant (one-way ANOVA using Tukey’s *post hoc* test). Tap: tracheal antimicrobial peptide. Lap: lingual antimicrobial peptide. The M scoring system is described in **Figure 1**.

Innate defense mechanisms in the skin of cattle remain largely unexplored despite being key to understanding the pathogenesis of DD. To elucidate the first line of innate effectors in the bovine skin, β-defensins, and cathelicidins were assessed in DD lesions of varying stages. Active M1 and M2 lesions had higher transcriptional gene expression of the β-defensin Tap when compared to normal (M0) healthy skin (**Figure 5A**). Transcriptional gene expression of the β-defensin Lap was not significantly altered during the course of DD although it tended to be higher in M1 and M2 lesions than in normal (M0) skin (*P* > 0.05) (**Figure 5B**). Transcriptional gene expression of Bmap-28, an important cathelicidin in cattle, was downregulated in active DD lesions (M1, M2, and M4.1) but did not differ from normal (M0) levels in the Tx-M2 lesions (**Figure 5C**).

### Cxcl-8, IL-10, and TLR4 Are Differently Expressed in Active and Chronic DD Lesions

**FIGURE 6:**
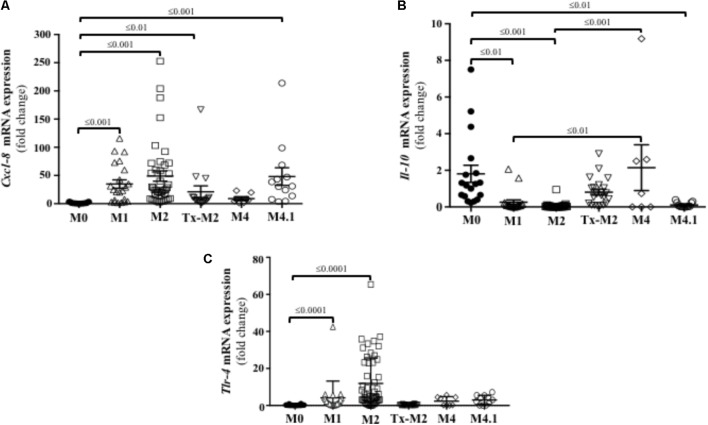
Transcriptional gene expression of cytokines and TLRs in foot lesions of cows at different M stages of digital dermatitis. Relative mRNA gene expression of Cxcl-8 **(A)**, IL-10 **(B)**, and TLR4 **(C)** was determined by RT-qPCR. Means + SEM are shown. Only significant comparisons (*P* < 0.05) are noted (one-way ANOVA using Tukey’s *post hoc* test). IL, interleukin. Tlr, toll-like receptor. The M scoring system is described in **Figure 1**.

Since active M2 lesions displayed marked infiltration of neutrophils (**Figure 3**), the presence of neutrophil chemoattractant factors was investigated. Directional movement of neutrophils into the skin is mostly regulated by C-X-C motif containing chemokine ligand 8 (Cxcl-8) (Kondo et al., 1993). Transcriptional gene expression of Cxcl-8 was upregulated in DD lesions at all stages except M4 (**Figure 6A**) and was highest in the active DD stages (M1, M2, and M4.1).

Transcription of IL-10 was downregulated in acute or reactivated DD stages (M1, M2, and M4.1), but returned to normal (M0) levels during the chronic M4 stage (**Figure 6B**). IFN-γ, which has pro-inflammatory and antiviral functions, was not expressed in the bovine DD lesions analyzed.

Both TLR2 and TLR4 are constitutively expressed in human keratinocytes and recognize lipoteichoic acid (Gram-positive bacteria) and lipopolysaccharide (Gram-negative bacteria), respectively (Mempel et al., 2003). Skin transcriptional expression of TLR4 was increased in the active DD (M1 and M2) stages in cattle (**Figure 6C**). No transcriptional gene expression of TLR2 was detected at any of the bovine DD lesions.

**FIGURE 7:**
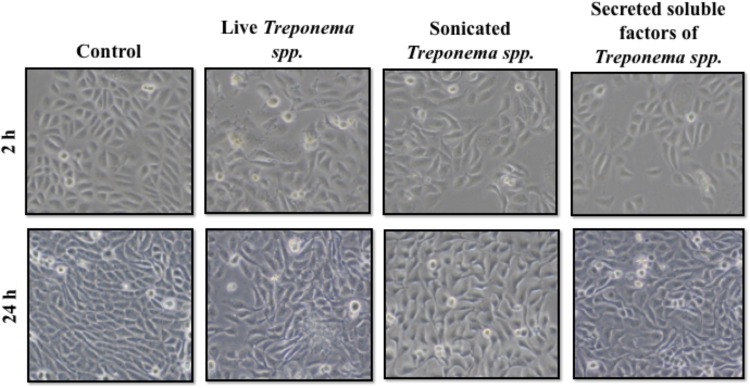
Morphology of human keratinocytes (HaCaT) after exposure to treponemes isolated from clinical cases of digital dermatitis in cows. HaCaT cells were incubated with control medium, live *Treponema* spp. (1 × 10^7^ CFU/mL), sonicated *Treponema* spp., and secreted soluble factors from *Treponema* spp. for 2 h **(Top)** and 24 h **(Bottom)** under aerobic conditions.

In cultured keratinocytes, treponemes induced early Cxcl-8 and TLR2 responses Keratinocytes exposed to live treponemes, lysed treponemes, or secreted soluble products from treponemes showed no changes in morphology or attachment upon exposure to treponeme forms for up to 24 h (**Figure 7**). Likewise, no effect of treponemes on HaCaT cell viability was shown by trypan blue staining.

**FIGURE 8:**
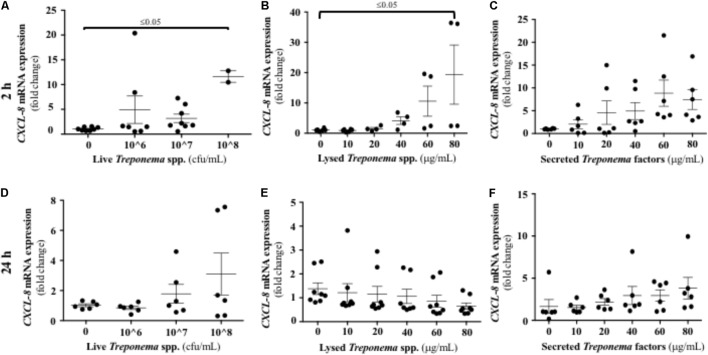
Transcriptional gene expression of Cxcl-8 in human keratinocytes challenged by *Treponema* spp. isolated from clinical cases of bovine digital dermatitis. Relative mRNA gene expression of Cxcl-8 was determined by RT-qPCR in HaCaT cells incubated with increasing concentrations of live *Treponema* spp. **(A,D)**, lysed *Treponema* spp. **(B,E)**, and secreted soluble factors from *Treponema* spp. **(C,F)** for 2 h (Top) and 24 h (Bottom) under aerobic conditions. Means + SEM are shown (*n* = 3 independent experiments run in triplicate). Only significant comparisons (*P* < 0.05) are noted (one-way ANOVA using Tukey’s *post hoc* test).

As neutrophilic infiltration and Cxcl-8 gene expression were increased in active bovine DD lesions, we investigated whether keratinocytes are a source of this neutrophil chemokine. Both live and lysed treponemes induced an early (2 h) increase in Cxcl-8 gene transcription levels in a dose-dependent manner in HaCaT cells (**Figures 8A,B**). Soluble factors secreted by treponemes also induced some Cxcl-8 gene expression after 2 h, but this was not statistically significant (*P* > 0.05) (**Figure 8C**). No differences in Cxcl-8 expression were found at 24 h post-challenge (**Figures 8D–F**). Thus, early Cxcl-8 synthesis in the skin was an epidermal response to treponemes isolated from bovine DD.

**FIGURE 9:**
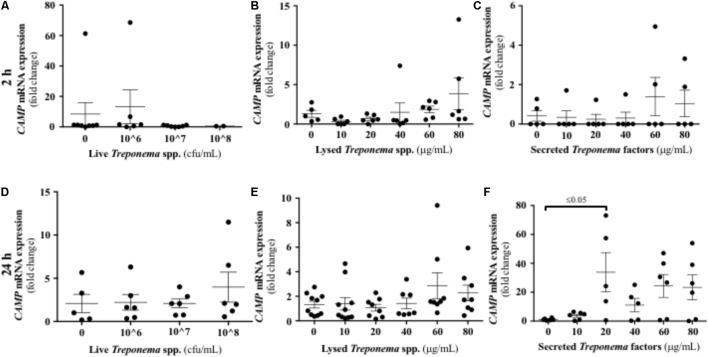
Transcriptional gene expression of cathelicidins in human keratinocytes challenged by *Treponema* spp. isolated from clinical cases of bovine digital dermatitis. Relative mRNA gene expression of cathelicidin CAMP was determined by RT-qPCR in HaCaT cells incubated with increasing concentrations of live *Treponema* spp. **(A,D)**, lysed *Treponema* spp. **(B,E)**, and secreted soluble factors from *Treponema* spp. **(C,F)** for 2 h (Top) and 24 h (Bottom) under aerobic conditions. Means + SEM are shown (*n* = 3 independent experiments run in triplicate). Only significant comparisons (*P* < 0.05) are noted (one-way ANOVA using Tukey’s *post hoc* test). CAMP, Cathelicidin antimicrobial peptide.

We evaluated the expression of CAMP, the only cathelicidin described in humans, in keratinocytes exposed to bovine treponemes. Neither live nor lysed treponemes increased CAMP gene transcription (**Figures 9A,B,D,E**). Only soluble factors secreted by treponemes promoted increased CAMP gene expression 24 h post-challenge (**Figures 9C,F**). Thus, in contrast to the early Cxcl-8 synthesis seen in response to treponemes, cathelicidins were only induced by factors released by treponemes after longer exposure.

**FIGURE 10:**
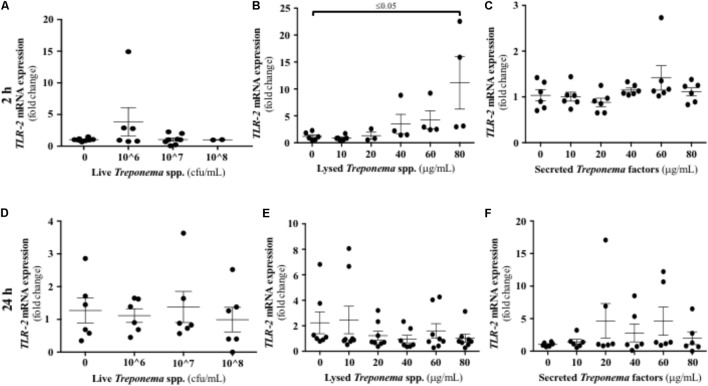
Transcriptional gene expression of TLR2 in human keratinocytes challenged by *Treponema* spp. isolated from clinical cases of bovine digital dermatitis. Relative mRNA gene expression of TLR2 was determined by RT-qPCR in HaCaT cells incubated with increasing concentrations of live *Treponema* spp. **(A,D)**, lysed *Treponema* spp. **(B,E)**, and secreted soluble factors from *Treponema* spp. **(C,F)** for 2 h (Top) and 24 h (Bottom) under aerobic conditions. Means + SEM are shown (*n* = 3 independent experiments run in triplicate). Only significant comparisons (*P* < 0.05) are noted (one-way ANOVA using Tukey’s *post hoc* test). TLR, toll-like receptor.

**FIGURE 11:**
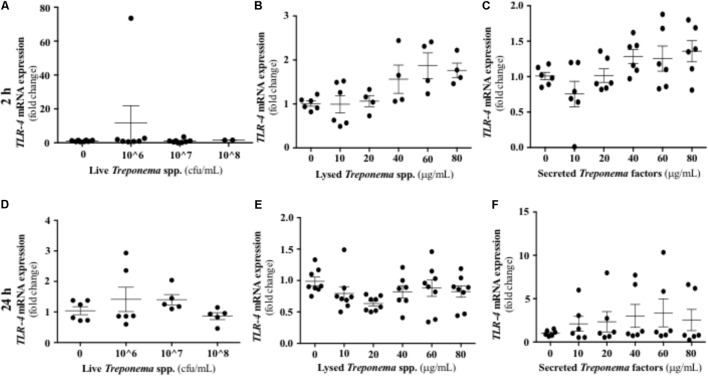
Transcriptional gene expression of TLR4 in human keratinocytes challenged by *Treponema* spp. isolated from clinical cases of bovine digital dermatitis. Relative mRNA gene expression of TLR4 was determined by RT-qPCR in HaCaT cells incubated increasing concentrations of live *Treponema* spp. **(A,D)**, lysed *Treponema* spp. **(B,E)**, and secreted soluble factors from *Treponema* spp. **(C,F)** for 2 h (Top) and 24 h (Bottom) under aerobic conditions. Means + SEM are shown (*n* = 3 independent experiments run in triplicate). Only significant comparisons (*P* < 0.05) are noted (one-way ANOVA using Tukey’s *post hoc* test). TLR, toll-like receptor.

Treponemes are spirochetes with structural components, such as peptidoglycan and lipoproteins, which can initiate TLR signaling (Nussbaum et al., 2009). In our study, neither live treponemes isolated from bovine DD lesions nor their secreted factors promoted a TLR2 response in HaCaT cells (**Figures 10A,C,D,F**). However, increasing amounts of lysed treponemes induced early (2 h), but not later (24 h), gene transcription of TLR2 in keratinocytes (**Figures 10B,E**). Regarding TLR4, whether treponemes in DD act through skin TLR4 for cellular attachment and invasion is unknown and perhaps unlikely as other treponemes, such as *T. pallidum*, lack LPS genes (Fraser et al., 1998). Our data suggest that this is the case, as TLR4 transcriptional gene expression was not significantly increased in keratinocytes at 2 h after exposure to either live or sonicated treponemes or their soluble secreted factors (**Figures 11A–C**). Likewise, TLR4 gene expression was not different from controls 24 h post-challenge (**Figures 11D–F**). These findings indicate that the keratinocyte innate response is limited to sensing structural treponeme components via TLR2 and rapidly producing the neutrophil chemoattract IL-8.

**FIGURE 12:**
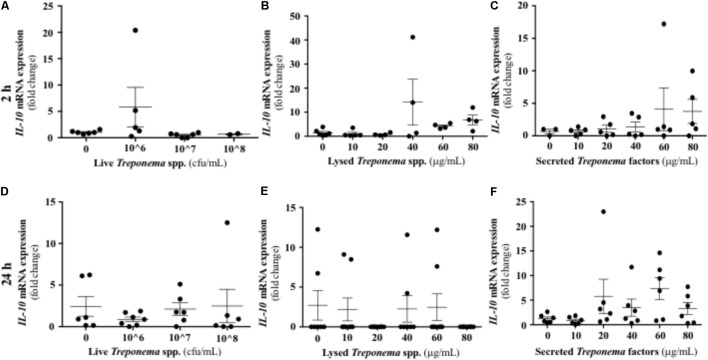
Transcriptional gene expression of IL-10 in human keratinocytes challenged by *Treponema* spp. isolated from clinical cases of bovine digital dermatitis. Relative mRNA gene expression of IL-10 was determined by RT-qPCR in HaCaT cells incubated with increasing concentrations of live *Treponema* spp. **(A,D)**, lysed *Treponema* spp. **(B,E)**, and secreted soluble factors from *Treponema* spp. **(C,F)** for 2 h (Top) and 24 h (Bottom) under aerobic conditions. Means + SEM are shown (*n* = 3 independent experiments run in triplicate). Only significant comparisons (*P* < 0.05) are noted (one-way ANOVA using Tukey’s *post hoc* test).

**FIGURE 13:**
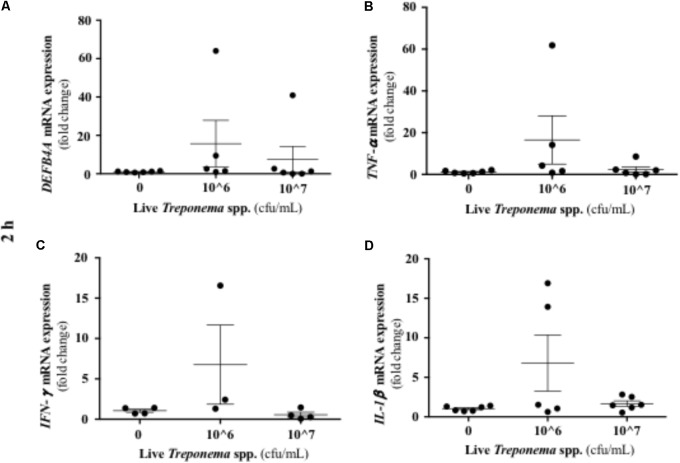
Transcriptional gene expression of β-defensins and pro-inflammatory cytokines in human keratinocytes challenged by *Treponema* spp. isolated from clinical cases of bovine digital dermatitis. Relative mRNA gene expression of DEFB4A **(A)**, TNF-α **(B)**, IFN-γ **(C)**, and IL-1β **(D)** was determined by RT-qPCR in HaCaT cells incubated with increasing concentrations of live *Treponema* spp. for 2 h under aerobic conditions. Means + SEM are shown (*n* = 3 independent experiments run in triplicate). Only significant comparisons (*P* < 0.05) are noted (one-way ANOVA using Tukey’s *post hoc* test). DEFB4A: Beta defensin 4A. TNF, tumor necrosis factor. IFN, interferon. IL, interleukin.

It has been established that IL-10 plays a role in suppressing inflammatory reactions, including those in certain skin disorders (O’Garra et al., 2004). In our study, cultured keratinocytes did not demonstrate substantial IL-10 gene expression after treponeme challenges (**Figures 12A–F**). Live treponemes isolated from bovine DD lesions did not modify β-defensin (DEFB4A) expression in keratinocytes (**Figure 13A**). Likewise, live treponemes did not promote significant transcriptional gene expression of the pro-inflammatory cytokines TNFα, IFN-γ, or IL-1β in HaCaT cell cultures (**Figures 13B–D**).

## Discussion

Using skin from cows with clinical DD and cultured human keratinocytes, we determined novel aspects of the innate immune response to *Treponema* spp. and the kinetics of local cytokine and host defense peptide expression in active and chronic bovine DD lesions. Active DD lesions (M1 and M2 and reactivated M4.1) were characterized by acute ulcerative inflammation, increased Cxcl-8, TLR4, and β-defensin gene expression, and the presence of abundant spirochetes. In contrast, chronic DD lesions (M4) were characterized by increased synthesis of anti-inflammatory IL-10, while lesions previously treated with the local antibiotic oxytetracycline (Tx-M2) had elevated cathelicidin expression. In the lesions of treated cows (Tx-M2), treponemes were detected by direct visualization but could not always be isolated in culture.

Our results indicate that infection with *Treponema* spp. induced Cxcl-8 expression in the bovine foot skin. The epidermis seems to be a main source of Cxcl-8 as live treponemes and their lysed bacterial cell structures also rapidly induced Cxcl-8 gene expression in cultured keratinocytes. It is possible that treponeme membrane components are major stimuli for the synthesis of epidermal Cxcl-8; the lipoprotein of *T. pallidum* has been previously shown to induce secretion of Cxcl-8 in vitamin D_3_-matured THP-1 cells (Sellati et al., 1998). The synthesis of epidermal Cxcl-8 in bovine skin infected with *Treponema* spp. could be crucial in the pathogenesis of DD. Keratinocytes in skin constitutively produce Cxcl-8 and its expression is increased in response to pro-inflammatory cytokines such as IL-1 and TNF-α (Kondo et al., 1993). In terms of function, Cxcl-8 is a chemokine with a strong ability to attract neutrophils and induce keratinocyte proliferation (Kolaczkowska and Kubes, 2013). Thus, Cxcl-8 production in active DD lesions may have a pronounced effect on the epidermal recruitment of neutrophils.

Our results suggest that synthesis of cathelicidins and β-defensins in bovine skin is part of the innate defense against DD. The protective effects of these peptides have been described in human skin keratinocytes, which constitutively produce β-defensin 1, and release β-defensin 2 in response to bacterial challenge or inflammation (Schneider et al., 2005). The importance of cathelicidins in the response to skin infection has been demonstrated in cathelicidin knockout mice (homozygous for null mutations in cathelicidins, *Camp^-/-^*); when challenged with *Staphylococcus aureus*, these mice developed more severe skin lesions than wild-type mice (Nizet et al., 2001; Nakatsuji et al., 2016). In cattle, previous studies on epithelial surfaces indicated β-defensin genes were mostly represented at the mucosa level by Tap and Lap (Cormican et al., 2008; Davies et al., 2008; Bagnicka et al., 2010). Those β-defensins have been described in bacterial infectious diseases of the gastrointestinal epithelium (Isobe et al., 2011) and the mammary epithelium (Isobe et al., 2009; Kosciuczuk et al., 2014). Moreover, Tap has been associated with early response to inflammation in the respiratory tract (Caverly et al., 2003). Likewise, BMAP-28 is a key bovine cathelicidin peptide with direct antimicrobial and immunomodulatory activity (Kosciuczuk et al., 2012). In our study, the expression of β-defensins varied among DD lesions, with different expression patterns during the active and chronic stages of the disease. The expression of the β-defensins Tap and Lap was higher in M1 and M2 lesions when the disease is active developing inflammation and treponemes are commonly present. In agreement, Tap expression occurs in early inflammation in the respiratory tract of calves under bacterial infection (Caverly et al., 2003). Our study revealed that elevated β-defensin transcription in bovine foot skin during active DD stages (M1 and M2) was associated with neutrophil infiltration and the synthesis of pro-inflammatory Cxcl-8. The synthesis of β-defensins could extend the lifespan of neutrophils within the DD lesions as β-defensins have been shown to suppress neutrophil apoptosis (Nagaoka et al., 2008). Additionally, β-defensins may aid in recruiting neutrophils to injury sites; human β-defensins 2 and 3 and their mouse orthologs, β-defensins 4 and 14, have been shown to interact with CCR2, a chemokine receptor expressed on monocytes, macrophages, and neutrophils (Rohrl et al., 2010). Thus, Tap and Lap could be established early in M1 lesions and remained throughout the development to M2 lesion. Both β-defensins might contribute to clearing bacterial infection via direct microbial killing or via recruitment of leukocytes during acute DD (M1 and M2).

In our study, expression of cathelicidins varied in the different stage DD lesions, suggesting differences in cathelicidin expression between the active and chronic stages of DD. The cathelicidin Bmap-28 was reduced to below basal levels of gene expression in active DD lesions (M1/M2), which suggests that treponemes may have some inhibitory effect on local cathelicidin synthesis. It has been similarly reported that *Salmonella enterica* serovar Typhimurium decreases the expression of host antimicrobial peptides in intestinal Paneth cells (Salzman et al., 2003). The levels of Bmap-28 were normal in antibiotic-treated DD lesions (Tx-M2). The reduction in Bmap-28 expression in active DD lesions with severe inflammation and its relatively greater expression in treated lesions suggests that Bmap-28 may be necessary for the protection of the skin against bacterial infection or for recovery of inflamed skin. Cathelicidins promote neutrophil influx to injury sites by chemoattraction (Kosciuczuk et al., 2012) or by inducing the expression of Cxcl-8 as demonstrated in human keratinocytes (Tjabringa et al., 2003). Cathelicidins can also contribute to skin ulcer repair by inducing keratinocyte migration via transactivation of the epidermal growth factor receptor (Tokumaru et al., 2005). Furthermore, cathelicidins could provide anti-treponeme effects during DD as these peptides have been shown to kill or block the infectivity of other treponemes, such as *T. pallidum* in a rabbit model (Cox et al., 2003). Interestingly, we found a lack of cathelicidin response in cultured keratinocytes exposed to *Treponema* spp. Cathelicidin expression only increased after incubation with high concentrations of the supernatant from cultured treponemes. Thus, cathelicidins seem to be important in reparative DD stages; however, they may be derived from leukocytes recruited later (e.g., macrophages) in the disease process or from keratinocytes after prolonged exposure to *Treponema* spp. or secreted products. It is noted that skin biopsies included infiltrating leukocytes, mostly in M2 lesions, which abundantly express host defense peptides and could contribute to the inflammatory and innate responses noticed in cattle skin lesions but not in the keratinocyte *in vitro* model.

Interleukin 10 is expressed by lymphocytes and neutrophils and negatively regulates the expression of pro-inflammatory cytokines; thus, it plays a role in resolving skin inflammation (Saraiva and O’Garra, 2010). In our study, transcriptional expression of IL-10 was suppressed in acute DD lesions (M1, M2, and recurrent stage M4.1) but was normal in chronic lesions (M4). The source of IL-10 in these cases could be infiltrating lymphocytes as we did not detect IL-10 in human keratinocytes exposed to *Treponema* spp. Lymphocytes (dermal CD4^+^ T cells) have been shown to be the main source of IL-10 in the Leishmaniasis-infected murine skin (Belkaid et al., 2001).

This study is one of the first to explore the innate immune mechanisms against *Treponema* spp. isolated from bovine DD. Our study demonstrates that the soluble and structural proteins of *Treponema* spp. are rapidly (in just 2 h) sensed by keratinocytes, and that detection of this pathogen occurs mostly via TLR2. Our findings using *Treponema* spp. isolated from bovine DD are similar to those of studies that used other treponemes. It has been shown that recognition of *T. denticola* occurs via TLR2 signaling in human gingival epithelial cells and macrophages (Asai et al., 2003; Ruby et al., 2007), whereas *T. brennaborense* requires both TLR2 and TLR4 to generate a signaling response in murine macrophages (Opitz et al., 2001). In our study, we found that TLR4 was present in the foot lesions of cattle with active DD (M1 and M2). The specific components of the *Treponema* spp. isolates that induce each TLR remain unknown, but studies have shown that either TLR2 or TLR4 may lead to activation of the MyD88 signaling pathway (Nussbaum et al., 2009). For instance, the major outer sheath proteins of *T. denticola* signaled via TLR2-MyD88 and lipooligosaccharide triggered a macrophage response via TLR4-MyD88 (Nussbaum et al., 2009). That this TLR2 upregulation was mediated by the components of killed treponemes rather than by live treponemes indicates that immune detection does not necessarily depend on active invasion by the live spirochete and that structural components of treponemes alone may be enough to induce an immune response. This is particularly pertinent to the development of inert vaccines containing treponeme factors that could induce TLR-dependent immune responses. Such innate immune defenses could aid in preventing invasion by inducing the synthesis of Cxcl-8 and recruitment of neutrophils. In addition, certain host defense peptides (β-defensins in active stages and cathelicidins in reparative stages) may influence the development of DD and promote wound healing. Although other microbial species were not identified in our collected skin biopsy samples, a recent deep sequencing analysis on collected DD samples (M0–M4.1) identified variable microbial communities during progression of disease (Krull et al., 2014). When compared to healthy control samples, microbial populations of early lesions were colonized by *Spirochaetaceae*, *Mycoplasmataceae*, *Moraxellaceae*, and *Porphyromonadaceae*. As the lesions progressed toward the M2 DD stage, the microbial population became predominated by Spirochaetaceae (Krull et al., 2014). Thus, other microbial components beyond *Treponema* spp. may be involved in the skin innate immunity in cattle with DD.

Macroscopic descriptions of the different stages of DD have been previously reported (Dopfer et al., 1997) and are commonly used as guidelines to identify the course of the disease in cattle, although the histologic changes associated with each DD stage are insufficiently described. In our study, clinical M stages and histopathologic findings were analyzed using a specific histologic grading scheme for DD (IDDSS) (Krull et al., 2016). The most severe dermatitis was present in M1 and M2 (acute) stages of DD with severe ulcerative and necrotic lesions and massive epidermal infiltration of neutrophils. Large amounts of treponemes were also revealed in M1 and M2 compared with other stages. Chronic M4 lesions were not necessarily identified as hyperkeratotic or proliferative as previously reported (Dopfer et al., 1997). In particular, Tx-M2 lesions varied in severity and type of inflammation and did not correspond to any specific histological pattern. This agrees with the previously reported finding that topical treatment with oxytetracycline was not conclusively curative and incomplete healing and lesion recurrence were common complaints (Berry et al., 2010). From a diagnostic point of view, different histological inflammatory responses existed within M stages. Although the clinical macroscopic M-system manifestation is an indication of the host innate immune response to DD, the IDDSS grade includes unique histopathological events, such as acute versus chronic inflammation characteristics and type of inflammatory cells. Thus, the macroscopic identification of DD lesions did not always accurately correlate with the expected histological description and host immune response. Given this clinical and histopathologic variation, DD appears to be a dynamic condition with acute and chronic phases that evoke progressive inflammatory and immunologic responses to the causative bacteria. In this scenario, the clinical M classification system may be an oversimplification that is unable to predict host responses and lesion outcomes reliably.

In summary, our work provides novel insights into the pathogenesis of DD in cattle. We have demonstrated progressive immunologic responses in bovine skin and keratinocytes infected with *Treponema* spp. The acute stage of inflammation was characterized by strong infiltration of neutrophils and Cxcl-8 and β-defensin synthesis, while cathelicidins expression was augmented in lesions treated with antibiotics. Exploring the innate factors involved in the pathophysiology and immune response to DD will allow the development of treatment alternatives that may decrease industry losses due to DD and will allow less reliance on conventional antibiotics and chemical footbaths.

## Author Contributions

KW and EC conceived and designed the experiments. KW, KO, and CB conducted the animal sampling. KW, CF, and PL carried out the *in vitro* experiments and analyzed the data. CB cultured and isolated treponemes from DD. RA carried out the immunohistochemistry images for *Treponema* identification. CK conducted the histopathological exams. KO, CK, CB, JDB, HB, and EC contributed to data analysis and interpretations as well as manuscript editing. KW, CK, and EC drafted and wrote the manuscript.

## Conflict of Interest Statement

The authors declare that the research was conducted in the absence of any commercial or financial relationships that could be construed as a potential conflict of interest.

## Footnotes

1 https://www.genenames.org/

## Supplementary Material

FIGURE S1 Identification of treponemes in foot lesions of cows with M2 stage of digital dermatitis. **(A,B)** Photomicrographs of *Treponema* spp. immune stained with a rabbit polyclonal antibody against a mixture of *Treponema* spp. isolated from foot lesions of cows with M2 digital dermatitis. Characteristic spirochete structures (arrows) were located in the superficial stratum of the skin or in the lumen. **(C)** Epidermal surface showing black-staining treponemes arranged individually or in clusters (arrows) throughout the superficial epidermis. Silver stain. Bar = 20 μm.
