# Supplementary figures and images for: A Differential Innate Immune Response in Active and Chronic Stages of Bovine Infectious Digital Dermatitis
Source: Front Microbiol. 2018 Jul 19;9:1586. doi: 10.3389/fmicb.2018.01586 (PMC6060252; doi:10.3389/fmicb.2018.01586)

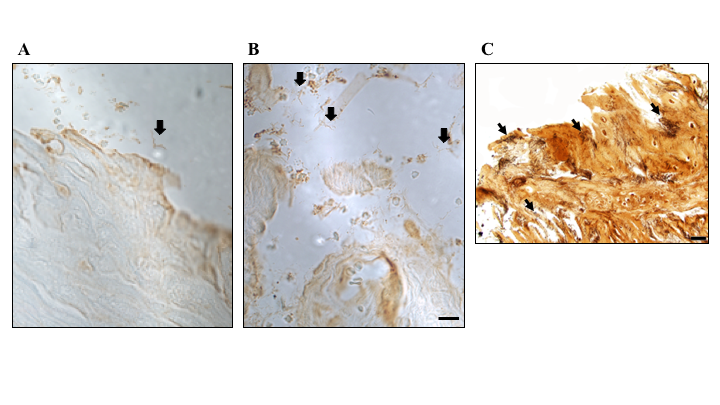

Supplement: FIGURE S1 — Identification of treponemes in foot lesions of cows with M2 stage of digital dermatitis. (A,B) Photomicrographs of Treponema spp. immune stained with a rabbit polyclonal antibody against a mixture of Treponema spp. isolated from foot lesions of cows with M2 digital dermatitis. Characteristic spirochete structures (arrows) were located in the superficial stratum of the skin or in the lumen. (C) Epidermal surface showing black-staining treponemes arranged individually or in clusters (arrows) throughout the superficial epidermis. Silver stain. Bar = 20 μm. [file Image_1.TIFF]
